# Reconstruction of metatarsal bone after giant cell tumor resection with no vascularized fibular graft in a pediatric patient: Case report and review of literature

**DOI:** 10.3389/fped.2022.970309

**Published:** 2022-10-12

**Authors:** M. Florio, S. Careri, C. Zoccali, A. G. Aulisa, F. Falciglia, R. M. Toniolo, M. Giordano

**Affiliations:** ^1^ Department of Surgery and Transplant, Division of Traumatology, Bambino Gesu’ Children Hospital (IRCCS), Rome, Italy; ^2^ Department of Human Anatomy, Histology, Forensic Medicine, Orthopedics, Sapienza University, Rome, Italy; ^3^ University of Cassino and Southern Lazio, Cassino, Italy

**Keywords:** pediatric giant cell tumor, foot tumors, metacarpal replacement, en-bloc resection, non-vascularized fibular graft

## Abstract

The Giant Cell tumor (GCT) is a benign, locally aggressive lesion that cause bone destruction and shows a malignant potential. It is a relatively common skeletal tumor that is therefore typically seen in young adults. Few cases are described in literature of GCT in the immature skeleton, and the metatarsal is an unusual location for a primary bone GCT, especially in pediatric age. Therefore, there are very few data reported regarding the management protocol of GCT in metatarsal bones. We report a case about the use of no vascularized fibular graft for an original Y-shaped reconstruction of the metatarsal bone after Giant Cell Tumor resection in a 9 years-old patient, and performed a literature review about metatarsal bone reconstruction in skeletally immature patient.

## Introduction

The Giant Cell tumor (GCT) is a benign, locally aggressive lesion that cause bone destruction and shows a malignant potential with occasional capacity to metastasize. Pulmonary metastases occur in about 2% of patients with GCT (1). It is a relatively common skeletal tumor that usually occurs in young adults, accounting for 4%–9.5% of all primary osseous tumors and 18%–23% of benign bone tumors (2, 3), but it can rarely occur also in skeletally immature patients (4). This tumor develops almost exclusively in the epiphysis of long bones, next to the adjacent joint. The most common location is around the knee region (distal femur, proximal tibia), but distal radius, fibular head, proximal humerus and sacrum can also be frequently involved (5). Its histogenesis is unclear. It presents a typical, but not specific, microscopic structure that justify the different terms used in the past to identify it: myeloid sarcoma, tumor of myeloplexus, osteoblastoclastoma and osteoclastoma. It contains a prominent and diffuse osteoclast-type giant cell component, uniformly distributed in a population of mononuclear plump epithelioid or spindle cells (6–8). The metatarsal is an unusual location for a primary bone GCT, especially in the immature skeleton. We report a case about the use of fibular graft for the reconstruction of the metatarsal bone after Giant Cell Tumor resection in a pediatric patient and performed a literature review about metatarsal bone reconstruction in skeletally immature patient.

## Methods and case description

A review of the literature was performed using common databases (Pubmed, Google Scholar), searching for “metatarsal reconstruction, pediatric giant cell tumor, en-bloc resection, non-vascularized fibular graft”. The literature research was focused on cases of GCT of metatarsal bone in pediatric age treated with wide resection and reconstruction with free fibular autograft.

A 9-years-old male, with a history of leukemia, presented to our hospital with a localized pain and a 3 cm area of swelling of the upper part of the right forefoot, first noticed 3 months earlier. There was no history of trauma, fever or any symptoms influencing his general health. Pain also got worse by walking. On examination, there was a fixed swelling area occupying the dorsal and the inner side of IV metatarsus, which was firm to hard consistency. There were no other specific abnormal findings on either of his feet on standard physical examinations.

## Diagnostic assessment

**Figure 1:**
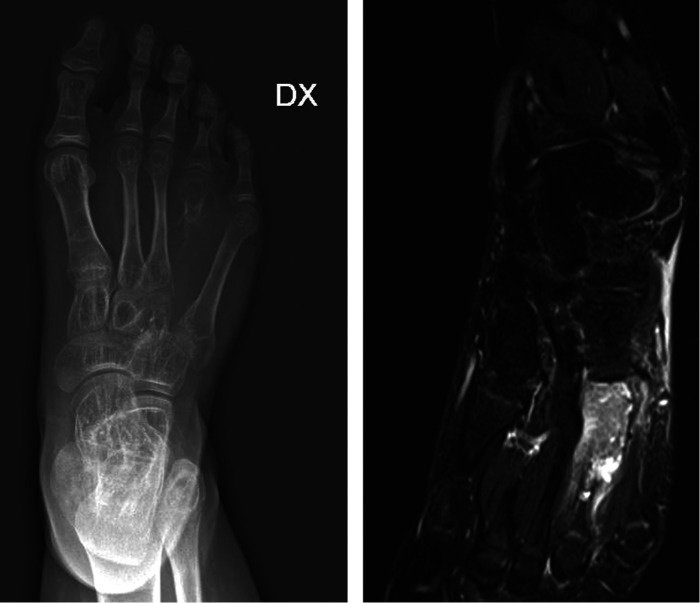
Pre-operative x-ray A/P (**A**) and MRI (**B**) view showing an expansile, lytic, encapsulated and iso-intense destructive lesion of the fourth metatarsus with soft tissue involvement.

**Figure 2:**
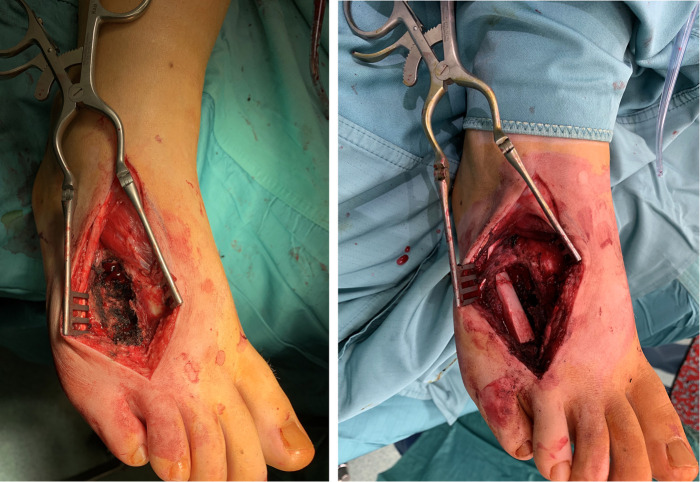
Intra-operative picture after en-bloc resection of tumor (**A**) and fibular grafting (**B**).

**Figure 3:**
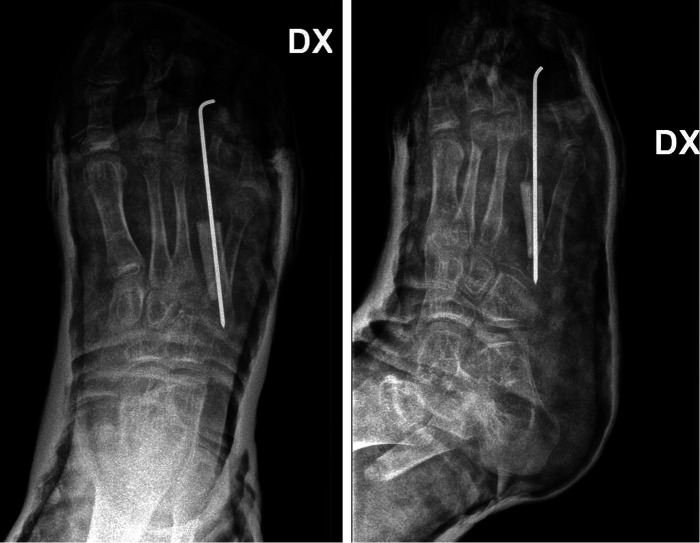
Post-operative x-ray A/P (**A**) and lateral (**B**) view showing reconstruction of IV metatarsal by fibular graft fixed to the base of V metatarsal (inverted Y shaped construct) using Kirschner’s wire and preserving tarso-metatarsal joint.

**Figure 4:**
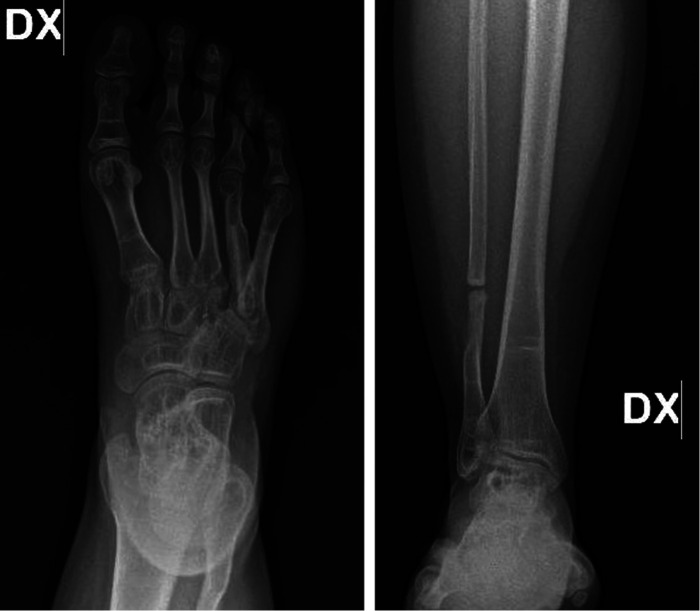
Follow-up x-ray of the foot and leg respectively taken at 12 months (**A**) and 24 months (**B**) showing incorporation of graft with no evidence of recurrences or other lesions and the donor site with an almost full recovery of the bone fibular gap.

The routine hematological examination was within normal limit. The patient was subdued a standard x-ray (Figure 1A) of the right foot, that have highlighted the presence of an expansile, osteolytic lesion, which expands and destroys the overlying cortex, without periosteal reaction, involving the base and the middle third of the IV metatarsus and extending into the soft tissue. Distally the growth plate appeared disrupted. Magnetic resonance imaging (Figure 1B) was suggestive of GCT and the biopsy confirmed the diagnosis. Chest x-rays ruled out lung metastasis. The patient underwent wide local resection, local adjuvant phenol therapy and a Y-shaped reconstruction with a no vascularized fibular autograft. The dorsal approach was used for the resection with incision including biopsy track (Figure 2A). The length of the required fibular graft was estimated preoperatively, and it was osteotomized out from the distal part of the ipsilateral fibula (Figure 2B). Fibula periosteum of the donor site was maintained intact and closed at the end of the harvest. The bone graft was synthesized, using a percutaneous intramedullary Kirschner-wire, from the head of the IV metatarsus to the V metatarsus proximal metaphysis, obtaining an inverted Y shaped construct and maintaining the tarso-metatarsal joint function (Figures 3A,B). The V metatarsal proximal medial cortex was scraped to allow an adequate fitting of the graft and to enhance osteointegration. The IV to V metatarsus junction, blocked by the K-wire was also reinforced by a vicryl bone suture. The patient's postoperative course was unremarkable and he was discharged after 5 days from surgery. A cast was applied for 2 months and the Kirschner wire was removed 50 days after surgery, without anesthesia. The patient began to walk, increasing weight-bearing progressively on the affected leg with crutches 70 days after surgery and then progressed to full weight-bearing walk, without pain, 4 months after surgery and after a radiographic control. Clinical and radiographic follow-up was performed at 1, 3, 6, 12 and 24 months (Figures 4A,B). Bone union at the proximal and distal junction sites occurred in 6 weeks. There was no evidence of peroneal nerve injury and the patient has achieved a full recovery of functions and normal activities at 12 months from surgery. At two years clinical and radiographic follow-up the patient showed normal walking without pain, no local recurrences or metastases and evidence of new, almost complete, ossification of the fibular bone gap.

## Discussion

Our case represents a combination of unusual location and age-group for GCT. Very few cases are described in literature of GCT in the immature skeleton, with an incidence from 1.8% to 10.6% (9, 10). In that case, the tumor is commonly found in bone metaphysis (4). Moreover, Bone GCT is rarely found in the metatarsal bones (11–13), it has more aggressive behavior and tends to grow faster in the foot (or hand) than in other bones, especially in young patients (14). Therefore, there are very few data reported regarding the management protocol of GCT in metatarsal bones. The goals of therapy are eradication of the tumor, to prevent damage to adjacent joint, and to prevent local recurrence. Different surgical procedures are adopted to treat this tumor, from intralesional curettage and bone grafting to wide resection with or without the use of several local adjuvants (phenol, liquid nitrogen, methyl methacrylate, hydrogen peroxide and alcohol) in order to control recurrences (5). The choice of treatment depends on both the site and the type of lesion. In our case, the patient presented a post-operative diagnosis of high grade GCT of bone (Campanacci grade 3 tumor) (15) with a thinned and partially destroyed overlying cortex, therefore it was impossible to carry out an intralesional curettage and wide resection was the treatment of choice.

Wide local resection is associated with a lower recurrence rate (5, 16), but has greater morbidity and higher rates of surgical complications when compared with intralesional curettage. However, metatarsal involvement makes wide resection of the lesion difficult as there is a little space between the rays of the foot. Looking at Enneking staging system and foot compartments (17) a radical resection of an extraosseous metatarsal lesion is difficult to obtain. In that case, the use of local adjuvants associated with surgical en-bloc resection can represent an effective solution in order to avoid amputation and control recurrence rate, as reported in literature (2). There are very few data reported about the reconstruction of metatarsal bones after GCT resection. Treatments described in literature may vary from resection alone to graft reconstruction from iliac crest or fibula. Fixation techniques may include both K-wires and plates.

Sheridan et al. (18), in 2020, in their retrospective review of 10 pediatric cases with non-traumatic primary bone defects, demonstrated that the use of non-vascularized fibular autograft is successful in the reconstruction of large bone defects secondary to malignant or benign pediatric bone tumors, reporting the largest known series of malignant pediatric tumors treated with this technique to date.

Rengsen et al. (19), in 2013, described a case of GCT of the 2nd metatarsal in a 14 years old girl. After resection, the metatarsal was reconstructed with a non-vascularized fibular graft, fixed with a dynamic compression plate. The outcome was good.

Compared to Rengsen procedure, our technique allows to remove the k-wire without further surgery and anesthesia and with less risks of hardware infection. Moreover, the use of a K wire through the growth plate did not caused damages of the cartilage. As already demonstrated by Guzzanti et al. in 1994 (20) the passage of a small, smooth hardware across the physis, then removed, do not stimulate physeal growth disorders. The tarso-metatarsal joint was maintained intact, avoiding walking impairment. In order to obtain that, an inverted Y shaped construct was chosen. Despite it was not an anatomical construct, pediatric orthopedic surgeons are used to Y-shaped metatarsal as a variant of post-axial polydactyly. The original construct was so carefully planned to avoid walking impairment, tarso-metatarsal joint disfunction and, at the same time, further surgeries to remove hardwares. Furthermore, one of the advantages of this technique is the remodeling capacity of the fibula and its relative ease of harvest. The periosteum preservation and closure has allowed a recovery of the bone gap. The closed membrane creates a biological chamber that permits revascularization and produces growth factors. The membrane also gathers growth factors with osteogenic potential. Looking at similarities with Masquelet technique, it's important to underline that there isn't bone union in cases with induced membrane, like periosteum, removal (21).

## Conclusion

The proximal meta-epiphysis of the metatarsus is an unusual place for primary bone GCT and it is important to know atypical locations in order to perform a proper diagnosis and treatment, especially in young patients. GCT should be conceded in the differential diagnosis of destroying lesions in the immature skeleton. This case study assessed the good outcomes with no recurrences of metatarsal GCT treated with resection and original reconstructive technique that shows to be a valid surgical option in the treatment of this particular and difficult condition.

## Data Availability

The original contributions presented in the study are included in the article/Supplementary Material, further inquiries can be directed to the corresponding author/s.

## References

[1] Siebenrock KA Unni KK Rock MG. Giant-cell tumour of bone metastasising to the lungs. A long-term follow-up. J Bone Joint Surg Br. (1998) 80(1):43–7. 10.1302/0301-620X.80B1.0800043 9460951

[2] Manaster BJ Doyle AJ. Giant cell tumors of bone. Radiol Clin North Am. (1993) 31:299–323. 10.1016/S0033-8389(22)02859-7 8446751

[3] Mirra J.M. Bone tumors: Clinical, radiologic and pathologic correlations. 2nd ed. Philadelphia, PA: Lea / Febiger (1989).

[4] Hoeffel JC Galloy MA Grignon Y Chastagner P Floquet J Mainard L Giant cell tumor of bone in children and adolescents. Rev Rhum Engl Ed. (1996) 63(9):618–23. 8938873

[5] Bridge JA Neff JR Mouron BJ. Giant cell tumor of bone. Chromosomal analysis of 48 specimens and review of the literature. Cancer Genet Cytogenet. (1992) 58(1):2–13. 10.1016/0165-4608(92)90125-R 1728946

[6] Turcotte RE. Giant cell tumor of bone. Orthop Clin North Am. (2006) 37(1):35–51. 10.1016/j.ocl.2005.08.005 16311110

[7] Cavanna L Biasini C Monfredo M Maniscalco P Mori M. Giant cell tumor of bone. Oncologist. (2014) 19(11):1207. 10.1634/theoncologist.2014-0267 25378541 PMC4221376

[8] Huvos AG, editor. Bone tumors: Diagnosis, treatment,and prognosis. Philadelphia, PA: Saunders (1991); Stewart MJ. The histogenesis of myeloid sarcoma. Lancet (1922); 2:1106–8.

[9] Picci P Manfrini M Zucchi V Gherlinzoni F Rock M Bertoni F Giant-cell tumor of bone in skeletally immature patients. J Bone Joint Surg Am. (1983) 65(4):486–90. 10.2106/00004623-198365040-00009 6833323

[10] Schutte HE Taconis WK. Giant cell tumor in children and adolescents. Skeletal Radiol. (1993) 22(3):173–6. 10.1007/BF00206148 8480203

[11] Ly JQ Arnett GW Beall DP. Case 122: giant cell tumor of the second metatarsal. Radiology. (2007) 245:288–91. 10.1148/radiol.2451031776 17885197

[12] Aaron AD Kenan S Klein MJ Hausman MR Abdelwahab IF Lewis MM. Case report 810: giant cell tumor of the first metatarsal. Skeletal Radiol. (1993) 22:543–5. 10.1007/BF00209107 8272895

[13] Wang EH Arbatin JJ. Allograft reconstruction of a large giant cell tumor of the first metatarsal: a case report. Foot Ankle Int. (2008) 29:97–100. 10.3113/FAI.2008.0097 18275747

[14] Biscaglia R Bacchini P Bertoni F. Giant cell tumor of the bones of the hand and foot. Cancer. (2000) 88:2022–32. 10.1002/(SICI)1097-0142(20000501)88:9<2022::AID-CNCR6>3.0.CO;2-Y 10813712

[15] Campanacci M Bladini N Boriani S Sudanese A. Giant cell tumor of bone. J Bone Joint Surg Am. (1987) 69:106–14. 10.2106/00004623-198769010-00018 3805057

[16] Lausten GS Jensen PK Schiodt T Lund B. Local recurrences in giant cell tumour of bone. Long-term follow up of 31 cases. Int Orthop. (1996) 20(3):172–6. 10.1007/s002640050057 8832321

[17] Enneking WF. A system of staging musculoskeletal neoplasm. Clin Orthop Relat Res. (1986) 204:9–24. 10.1097/00003086-198603000-00003 3456859

[18] Sheridan GA Cassidy JT Donnelly A Noonan M Kelly PM Moore DP Non-vascularised fibular autograft for reconstruction of paediatric bone defects: an analysis of 10 cases. Strateg Trauma Limb Reconstr. (2020) 15(2):84–90. 10.5005/jp-journals-10080-1462 PMC7801896 33505524

[19] Rengsen P Tiong KL Teo YM Goh TC Sivapathasundram N. Reconstruction of the second metatarsal with non-vascularized fibular graft following en-bloc resection for giant celll tumour: a case report. Malays Orthop J. (2013) 7(3):15–7. 10.5704/MOJ.1311.001 25674301 PMC4322136

[20] Guzzanti V Falciglia F Gigante A Fabbriciani C. The effect of intra-articular ACL reconstruction on the growth plates of rabbits. J Bone Joint Surg Br. (1994) 76(6):960–3. 10.1302/0301-620X.76B6.7983128 7983128

[21] Careri S Vitiello R Oliva MS Ziranu A Maccauro G Perisano C. Masquelet technique and osteomyelitis: innovations and literature review. Eur Rev Med Pharmacol Sci. (2019) 23(2 Suppl):210–6. 10.26355/eurrev_201904_17495 30977888

